# Irritable bowel syndrome: new insights into symptom mechanisms and advances in treatment

**DOI:** 10.12688/f1000research.7992.1

**Published:** 2016-04-29

**Authors:** Robin Spiller

**Affiliations:** 1 Nottingham Digestive Diseases Centre, University of Nottingham, Nottingham, UK

**Keywords:** IBS, IBD, MRI studies, FODMAP diet, IBS treatment advances

## Abstract

Despite being one of the most common conditions leading to gastroenterological referral, irritable bowel syndrome (IBS) is poorly understood. However, recent years have seen major advances. These include new understanding of the role of both inflammation and altered microbiota as well as the impact of dietary intolerances as illuminated by magnetic resonance imaging (MRI), which has thrown new light on IBS. This article will review new data on how excessive bile acid secretion mediates diarrhea and evidence from post infectious IBS which has shown how gut inflammation can alter gut microbiota and function. Studies of patients with inflammatory bowel disease (IBD) have also shown that even when inflammation is in remission, the altered enteric nerves and abnormal microbiota can generate IBS-like symptoms. The efficacy of the low FODMAP diet as a treatment for bloating, flatulence, and abdominal discomfort has been demonstrated by randomized controlled trials. MRI studies, which can quantify intestinal volumes, have provided new insights into how FODMAPs cause symptoms. This article will focus on these areas together with recent trials of new agents, which this author believes will alter clinical practice within the foreseeable future.

## Introduction

Irritable bowel syndrome (IBS) is one of the most common gastroenterological diagnoses, experienced by around 11% of the population. Symptoms consist of abdominal pain associated with erratic bowel habit and variable changes in stool form and frequency, suggesting considerable heterogeneity in underlying mechanisms. Despite IBS’ high prevalence, these mechanisms are poorly understood and treatment is unsatisfactory. This important unmet clinical need is a very active research area. This article will focus on recent significant advances, which this author believes are likely to influence clinical practice within the foreseeable future. These include better understanding of the role of bile acids in causing diarrhea/constipation, significance of alterations in gut microbiota, alterations in enteric innervation and serotonin availability associated with inflammation, IBS-like symptoms in patients with quiescent inflammatory bowel disease (IBD), and several new treatments including the low-FODMAP diet, guanylate cyclase C activators, and 5-hydroxytryptamine 3 (5-HT3) receptor antagonists.

## New insights into mechanisms of IBS symptoms

### Bile acids and bowel dysfunction

The laxative properties of bile acids have been recognized since 1868, when ‘liver’ pills composed of ox bile were patented and widely promoted as panacea supplements. Scientific study had to wait until the 1970s, when perfusion studies showed that bile salts stimulated enterocyte secretion and in excess caused diarrhea. Initially, terminal ileal resection was identified as the commonest cause of bile acid diarrhea, but it is now recognized that the negative feedback loop controlling hepatic bile acid production may be disturbed in patients with intact intestines. Bile acids absorbed in the human ileum activate the nuclear receptor farnesoid X to stimulate the production of fibroblast growth factor 19 (FGF19). This circulates to the liver where, acting via the FGF receptor 4, it inhibits cholesterol 7-hydroxylase (CYP7A1), the rate-limiting enzyme that converts cholesterol to 7α-Hydroxy-4-cholesten-3-one (C4), an intermediate step in the production of cholic and chenodeoxycholic acid. Decreased circulating FGF19 leading to excessive production of bile acids can be primary or secondary to bile salt malabsorption caused by ileal resection or ileitis. If excessive bile acids enter the colon, they stimulate colonic secretion and increase stool water. A meta-analysis suggests that 10% of patients with IBS with diarrhea (IBS-D)-like symptoms have severe bile acid malabsorption, with <5% retention at 7 days ^1^. A recent survey in the UK suggests that bile acid diarrhea accounts for nearly one in four of IBS patients referred to secondary care with diarrhea ^2^.

Identifying patients with overproduction of bile salts used to depend on demonstrating reduced 7-day retention of an artificial radiolabeled bile acid, selenium-75 homocholic acid taurine (SeHCAT), the normal being >15%. Retention of <5% is associated with a 96% response to cholestyramine ^1^, while lesser degrees of malabsorption produce less favorable results, with only 37% responding who have SeHCAT values >5% but <10% ^3^. Access to SeHCAT is limited worldwide, so the recent demonstration that a fasting FGF19 <145 pg/ml predicts SeHCAT <10% with a negative predictive value of 82% and a positive predictive value of 61% ^4^ suggests an alternative, which, as it is an enzyme-linked immunosorbent assay (ELISA), could be widely used unlike older HPLC methods. Newer, more convenient assays for C4 are also being developed. What causes a low FGF19 level in each case remains to be determined, but some cases of bile acid malabsorption begin acutely after an episode of ileitis, which is a common feature of both *Salmonella* species and *Campylobacter jejuni* gastroenteritis. Sudden onset associated with high-volume nocturnal diarrhea are characteristic features ^5^.

The laxative effects of bile acids has been exploited by inhibitors of bile acid uptake such as elobixibat, which reduce FGF19, increase bile acid synthesis, and have been shown in phase II studies to be effective treatments for constipation ^6, 7^.

The variability in symptoms with bile acid diarrhea suggests individual differences in sensitivity to bile acids. A single nucleotide polymorphism, rs11554825, in the membrane-bound bile acid receptor TGR5 (G-protein-coupled bile acid receptor 1, also known as GpBAR1) has been suggested to be linked to small bowel and colonic transit, which were faster with TT versus TC/CC variants ^8^. Further, more detailed studies in a smaller group showed faster colonic transit with both TT and CC TGR5 variants, possibly due to an interaction with klotho β (KLB) ^9^. However, more work is needed as these studies are underpowered and the functional significance of the rs11554825 variants in TGR5 has yet to be established.

### IBS in IBD

IBD, particularly Crohn’s disease, can mimic many IBS symptoms during acute inflammatory flares, but it is increasingly recognized that acute inflammation leaves persistent changes in both nerve and muscle, which leads to IBS-like symptoms, even during remission ^10, 11^. Occult inflammation can be detected with increases in fecal calprotectin in some cases ^12^, but that still leaves around one-third with IBS-like symptoms ^13^. The underlying mechanisms may include altered permeability and ongoing low-level immune activation, as has been shown in the cecal biopsies of IBD patients in apparent remission but with IBS symptoms ^14^. Other possible mechanisms include persisting alterations in enteric nerves and serotonin signaling (see below). The importance here is to recognize that such symptoms may respond better to IBS treatment including dietary restrictions rather than increasing immunosuppression with all of its inherent risks.

### Changes in enteric nerves

Several recent studies have examined mucosal innervation in IBS and found increases in nerves expressing the transient receptor potential vanilloid channel (TRPV1) ^15^, a peptide associated with pain pathways which also plays a key role in mechanosensitivity ^16^. TRPV1 is upregulated by inflammation and has been shown to be increased in the rectosigmoid mucosa of IBD patients who continue to experience pain despite apparent disease quiescence ^17^. Proximity of activated mast cells to enteric nerves has been shown to correlate with severity of abdominal pain in IBS ^18^, and more recently a study of 101 IBS patient biopsies has shown increased amounts of neural tissue and increases in the growth-associated protein 43 (GAP43). Furthermore, biopsy supernatants increased neurogenesis in primary culture of enteric neurons ^19^. Whether this stimulation of nerve growth causes the close association of enteric nerves and mast cells and contributes to visceral hypersensitivity in IBS remains to be determined.

## Alterations of serotonin transporter

The action of 5-HT at the synapse is terminated by active reuptake of 5-HT by the serotonin transporter (SERT). Several studies in IBS patients have suggested impairment of SERT in both platelets ^20^ and duodenal mucosa ^21^, though the evidence in the colon is contradictory, with some reporting a decrease ^22, 23^ and others no change ^24^. Many such mechanistic studies use small numbers of patients, so, given the heterogeneity of IBS, conflicting results are not unexpected. The existence of subgroups of patients with abnormally increased or decreased mucosal 5-HT means that while some will respond to 5-HT receptor antagonists, others need 5-HT agonists. A polymorphism in the promoter region of the SERT gene alters SERT efficiency with the long form *ll* increasing efficiency and being associated with IBS with constipation (IBS-C), while the short form *ss* is increased in IBS-D ^25^. Genetic differences in tryptophan hydroxylase-1 enzyme (TPH-1), the rate-limiting step in 5-HT synthesis, has been reported to predict response to 5-HT3 receptor antagonists ^26^. Similarly, the SERT promoter polymorphism has been reported to predict response to alosetron in IBS-D with *sl* genotype showing reduced responsiveness ^27^, but this was not confirmed in a larger trial with ondansetron ^28^.

### New insights from magnetic resonance imaging of the colon and small intestine

Magnetic resonance imaging (MRI) provides a unique opportunity to image the undisturbed gut, since by using a range of sequences adequate contrast can be obtained with normal gut contents ^29^. Such studies have provided for the first time accurate assessments of small bowel and regional colonic volumes in normal subjects. The resting small bowel contains surprisingly little free water, varying in different studies from 50 ml ^30^ to 150 ml ^31^. This rises rapidly to around 400 ml after an osmotic stress such as that provided by mannitol ^30^ or fructose ^32^ or falls when readily absorbable fluids are provided such as sucrose and glucose ^30^. High-fat meals, by contrast, cause a rapid rise in small bowel water content probably due to stimulation of pancreaticobiliary secretions ^33^.

**Figure 1.**
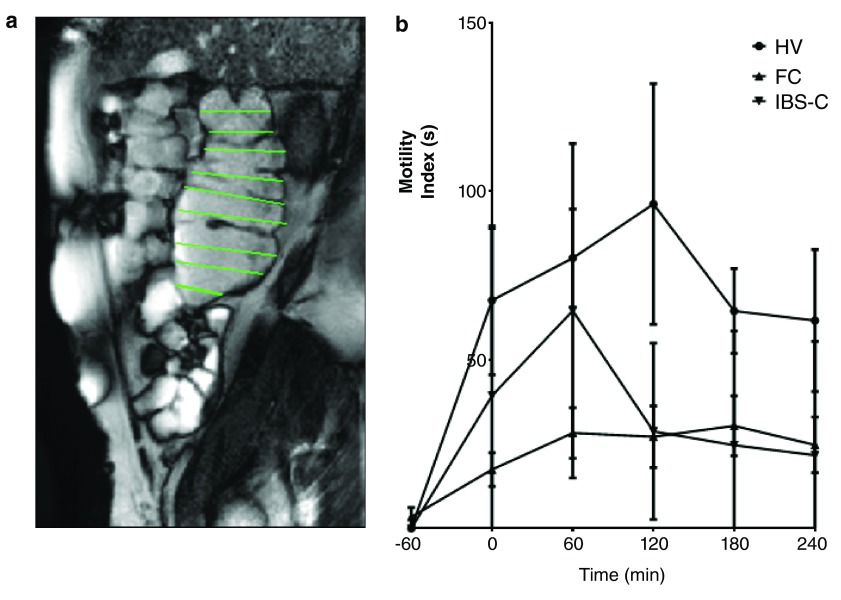
( **a**) Sagittal magnetic resonance image of ascending colon taken during cine recording. A system of image registration removes the movement due to diaphragmatic movements during respiration. The operator draws lines at right angles to the colonic axis and these lines are automatically propagated through the cine series. The change in line length between time points gives the transverse wall velocity and the motility index (MI) = % of lines at all time points in which the change in transverse wall velocity is >0.5 mm/s. ( **b**) Motility index of the ascending colon following ingestion of 1L of the osmotic laxative Moviprep commencing at time -60 minutes. This shows the normal rapid increase in motility in healthy volunteers (HV) with markedly impaired response in patients with functional constipation (FC). Irritable bowel syndrome with constipation (IBS-C) patients showed an initially normal response which had faded by the second hour. Data from Lam *et al.* ^34^.

MRI has shown that there is a very wide normal range of colonic volumes with the ascending colon being mean (standard deviation [SD]) 203 (75) ml, transverse colon 232 (100) ml, and descending colon 151 (71) ml, with total colonic volumes being 632 (167) ml. While IBS-D patients have fasting colonic volumes within the normal range, those with functional constipation have significantly increased ascending colon and total colon volumes at 597 (170) and 1505 (387) ml, respectively ^34^. Interestingly, by contrast, 95% of patients with IBS-C had colonic volumes within the normal range. In addition to fasting scans, it is simple to assess the response to feeding and also to a stronger stimulus provided by the osmotic laxative Moviprep ^R^ using cine MRI. This shows marked impairment of colonic motility in functional constipation but not IBS-C ( Figure 1) ^34^. The technique can also be used to show the mode of action of therapeutic agents including Movicol, loperamide, ondansetron, and ispaghula and could be useful in the future when screening drugs designed to alter colonic transit ^29^.

### Dietary intolerances

Many IBS patients report that their symptoms are aggravated by eating certain foods, and several uncontrolled studies found 36–40% of patients could be helped by selective exclusion of a range of foods often including dairy, wheat, onions, and fruit ^35, 36^. Double blind exclusion diets are very demanding and few have been done until recently, when a clearer definition of what was being excluded was developed based on the FODMAP concept ( Text Box 1).

**Table:** 

FODMAP concept	
• **F**ermentable • **O**ligosaccharides • **D**isaccharides • **M**onosaccharides • **A**ND • **P**olyhydric alcohols	- FOS, GOS, fructans, raffinose, inulin - lactose (galactose-glucose) - sucrose (glucose-fructose) - fructose - sorbitol, mannitol, sylitol, maltitol
Key features are poor absorption in the small bowel and rapid fermentation in the colon	

### FODMAP reduction to control IBS symptoms

Systematic application of this diet required the chemical analysis of common foods to identify those that contained significant amounts of these substances. The pioneers were the group in Monash University led by Gibson, a gastroenterologist, and Muir, a biochemist. The most important sources of FODMAPs in the western diet are wheat, onions, and fruit with an excess of fructose over glucose such as apples and pears. Dairy products are also important in those with lactose malabsorption. The first rigorous placebo-controlled diet published in 2014 showed that the low-FODMAP diet improved bloating and abdominal pain/discomfort when compared to the standard Australian diet ^37^. It should be noted, however, that the low-FODMAP diet does not alter bowel habit consistently and so cannot be expected to benefit those whose main problem is diarrhea or constipation. The low-FODMAP diet has been compared with an ‘IBS diet’ in a randomized trial that showed a similar improvement in symptoms ^38^. There is some overlap between the two diets, but the ‘IBS diet’ gives more general advice like regular meals and exercise along with avoiding foods thought to promote gas formation without the rigor of a diet based on the chemical composition of food. The complexity of the FODMAP diet makes it difficult to implement, an obstacle which perhaps could be overcome by excluding just the major sources of FODMAPs in any individual’s diet (e.g. wheat, onions, and dairy) and not bothering with items which individually contribute only small amounts or dairy products in those with the lactose persistence genotype ^39^.

### Changes in microbiota

**Figure 2.**
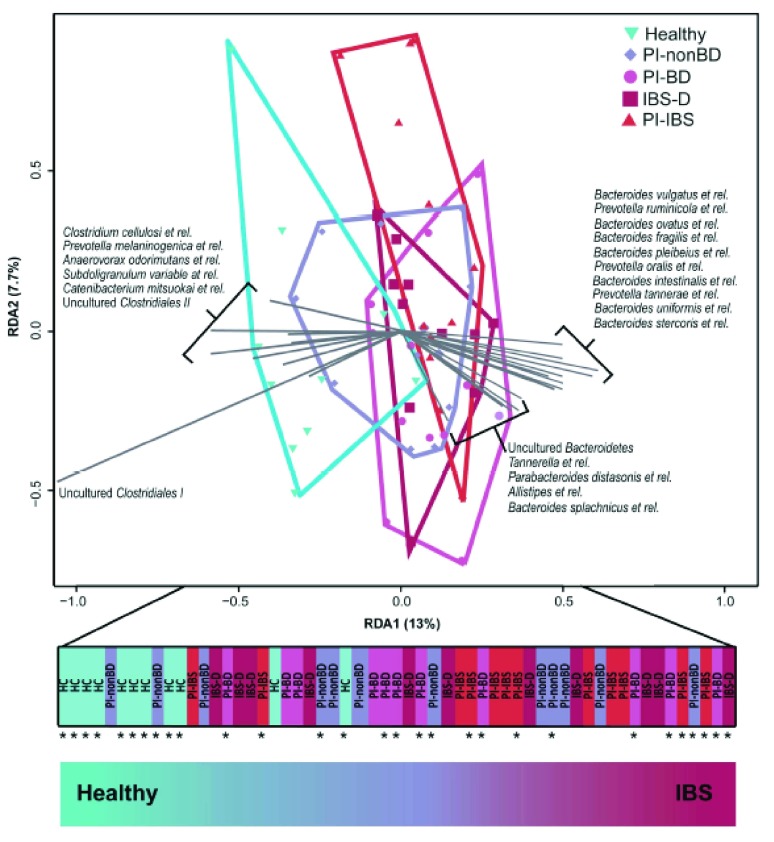
Redundancy analysis using 27 genus-like taxa to separate 1) healthy controls from 2) individuals who had had Campylobacter but recovered with no bowel disturbance (PI-nonBD), 3) individuals who had had Campylobacter but with persistent bowel disturbance (PI-BD), 4) individuals with post infectious IBS (PI-IBS), and 5) individuals with IBS with diarrhea (IBS-D). The primary axis was used as an index of dysbiosis, which separates these groups in a graded fashion from health to disease. Reproduced from Jalanka *et al.* ^45^.

The advances associated with non-culture-based methods of microbiota assessment using ribosomal RNA analysis has led to numerous studies of the microbiota in IBS with somewhat conflicting results (for review, see 40). This is perhaps not surprising, since it is clear that diet and transit are both major determinants of microbiota composition and most studies of the microbiota in IBS have failed to control for these factors ^41^. Fast transit, such as is seen in IBS-D patients, gives a survival advantage to either organisms that multiply rapidly or those that adhere well to the mucosa ^42^. This latter study showed that stool consistency was an important predictor of enterotype but did not comment on how consistent this was within an individual. One of the key features of IBS is the erratic pattern of stool form ^43^, with both hard and loose stool within a time period as short as 24 hours, suggesting that stool microbiota might also be unstable in IBS. A useful study demonstrated that while a subgroup have microbiota that are distinctly different from normal, many IBS patients have microbiota that cluster with normal controls. Interestingly, the group with ‘normal’ microbiota were more likely to have clinically significant depression, suggesting that abnormal microbiota might represent a group with a predominantly peripheral gut abnormality, while normal microbiota might be a signature of those in whom the main abnormality lies centrally ^44^. Acute gastroenteritis causes a marked disturbance of the gut microbiota with overgrowth of pathogen and a marked reduction in diversity. This is followed by a return towards the former equilibrium but, as recently shown, distinct differences remain after *Campylobacter jejuni* enteritis, the commonest cause of food poisoning in the UK. Restricting analysis to just 27 genus-like taxa, we showed in a redundancy analysis that the individuals could be ordered on the primary axis from health at one end to IBS at the other with infected but recovered individuals in between ^45^ ( Figure 2).

### Evidence of abnormal small bowel microbiota

This is an area of considerable confusion, largely because there is a gradient of bacterial density within the small bowel ranging from 10 ^-1^ ml in the duodenum to 10 ^7^ ml in the terminal ileum. Identifying an abnormal increase in luminal bacteria requires defining this gradient throughout the small bowel, something which current tests cannot do. Intubation and analysis of aspirates give a single value, usually from the jejunum. Using such a test, values exceeding the accepted threshold of 10 ^5^/ml are found in around 4% of both controls and IBS patients ^46^. Lactulose breath hydrogen tests (LBHTs) alone are impossible to interpret, as one can rarely exclude the possibility that any early rise in breath hydrogen observed is due to lactulose reaching the cecum. However, this can, to some extent, be overcome by combining with scintigraphic assessment of orocecal transit (SOCT), which gives a time when >5% of isotope has entered the colon. If the breath hydrogen rise occurs before this time then it is usually interpreted as showing abnormal microbiota in the small bowel. However, it should be noted that this requires that breath hydrogen would not rise if <5%, i.e. 0.5 g, had entered the colon, which is an unproven assumption, as this amount of lactulose will produce 16 ml of hydrogen ^47^, which, depending on the rate of excretion, could raise breath hydrogen by much more than the 10 parts per million required for a positive test. The terminal ileum is an area where the stasis which favors growth of colonic anaerobes is often observed, especially during fasting ^48^, so this is most likely to be the site where microbiota would proliferate. A recent study using this technique suggested that around one-third of IBS patients attending a Chinese outpatient clinic have a positive LBHT/SOCT, though it is unclear how these were selected or whether this finding is generalizable to other clinics. A positive test did seem to predict a better response to rifaximin, but these studies need repeating as the numbers were very small ^49^. These values are comparable with a previous large study using the glucose breath hydrogen test, which reported positive tests in 31% of 65 IBS patients versus 4% in 105 healthy controls ^50^.

### Biomarkers of disease mechanism

Where the history is confusing, colonic transit may be helpful in predicting response to drugs accelerating or retarding transit ^51^. Low SeHCAT values predict response to cholestyramine, but otherwise there are currently few other biomarkers in clinical use that can predict response to specific therapies. Measures of visceral sensitivity do not appear to predict response to ketanserin, even though this did increase the threshold for discomfort during a barostat study ^52^. Genetic tests might fare better, but preliminary reports suggesting TPH-1 polymorphisms predict response to ramosetron need confirmation in larger studies ^26^. Genetic testing for lactose intolerance could replace LBHT, being more convenient and highly sensitive ^53^. Though interesting mechanistically, the mucosal biopsy assays including histology and mediator release show such wide variability that none so far are useful diagnostically nor in predicting treatment response.

## Advances in treatment for IBS

### Opioids for IBS-D

Loperamide is a safe and effective anti-diarrheal agent, which has been shown in randomized controlled trials (RCTs) to reduce bowel frequency in IBS-D but with little benefit on pain ^54^, which may actually increase ^55^. Despite this, loperamide improves quality of life since it allows planning of trips and socializing, which anxious IBS-D patients often avoid for fear of fecal urgency or even incontinence. More recently, eluxadoline, a mu-opioid receptor agonist with delta-opioid receptor antagonistic action, has been shown in a large, high-quality phase IIb dose-finding RCT to benefit IBS-D with a 14% increase in responder rates (28% versus 14%) after 12 weeks of either 100 or 200 mg twice daily ^56^. However, this mode of action does carry a risk of causing acute pancreatitis through sphincter of Oddi spasm, which may prove unacceptable in IBS.

### 5-HT3 receptor antagonists for IBS-D: alosetron, ramosetron, and ondansetron

**Figure 3.**
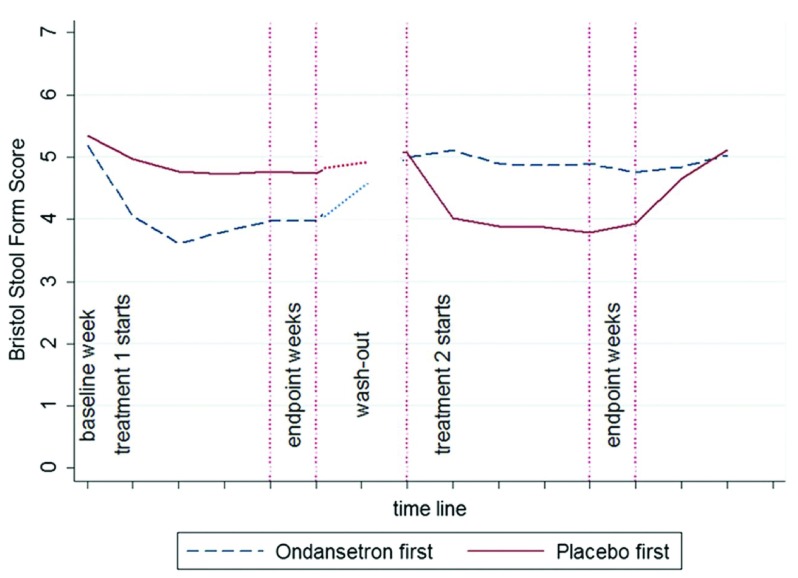
The time course of stool consistency during randomized cross-over. Stool form score fell into the normal range 3–5 within 1 week of starting ondansetron, rapidly returning to baseline on discontinuation. There was very little placebo response. Reproduced from Garsed *et al.* ^28^.

5-HT3 receptor antagonists (5-HT3RAs) are effective treatments for IBS-D ^57^, slowing transit, reducing bowel frequency, normalizing stool consistency, and reducing urgency ^58^, which is one of the key symptoms that impair quality of life in IBS-D. Alosetron has been shown to significantly improve quality of life ^59^. Constipation is a common side effect, which was reported in around 25% of those given standard doses of alosetron. While this can be controlled by dose reduction, this is not true of ischemic colitis, a much rarer side effect, which was seen in around one in 700 patients ^60, 61^. Although not life threatening, ischemic colitis led to alosetron’s withdrawal from general marketing, though now it is off patent, use may increase. Ramosetron is effective at a very low dose, 5 µg in men ^62, 63^ and 2.5 µg in women ^64^, with an acceptably low rate of constipation and no reports of ischemic colitis. Recently, the much less potent 5-HT3RA ondansetron, given at a dose of 4 mg, range 2–6 mg, was shown to be highly effective at improving stool consistency ( Figure 3), reducing stool frequency and reducing urgency, with 70% reporting adequate relief of IBS symptoms on ondansetron compared to 16% on placebo, giving a number needed to treat (NNT) of two ^28^. It is worth noting that ondansetron has been used for over two decades with no reports of ischemic colitis and has an excellent safety record, a feature which is so important for IBS medication.

### Secretagogues

Recently, two new secretagogues, lubiprostone and linaclotide, have been introduced for the treatment of both chronic constipation and IBS-C. Both are supported by large RCTs performed to high standards ^65– 68^. Lubiprostone stimulates chloride secretion by activating the type 2 chloride ion channel ^69^. It does not alter pain thresholds during rectal distension using a barostat ^70^ but does accelerate transit in healthy volunteers ^71^. It relieves overall IBS-C symptoms with 17.9% responder rate compared to 10.1% with placebo and this is associated with modest reductions in pain and straining ^65^. This gives a NNT of 13. Around 25% of patients experience nausea, but only around 5% discontinue its use because of this. Linaclotide also stimulates chloride secretion by activating the guanylate cyclase C receptor, increasing cyclic guanosine monophosphate (cGMP), an important intracellular second messenger that activates the cystic fibrosis transmembrane conductance regulator (CFTR) to cause chloride secretion ^72^. Linaclotide, 290 µg daily, improved both pain and stool consistency in a 12-week trial of 800 IBS-C patients, meeting the FDA guidelines in 33.6% compared to 21% on placebo, giving an NNT of eight ^73^. The benefit persisted in another 26-week trial ^67^. Both trials showed improvement in stool consistency and frequency within the first week, while abdominal discomfort continued to improve over a period of about 6 weeks. The main side effect is diarrhea, experienced in 19.7%, severe in 2%, and leading to drug discontinuation in 4.5% ^6^.

## Future directions

The heterogeneity of IBS means that future studies must aim to be larger and more mechanistic than in the past. There are too many small studies performed by isolated research groups with contradictory conclusions. These will be resolved only by larger multicenter studies involving consortia of researchers from many different areas. Such studies should include not only symptom responses but also measurement of relevant biomarkers, which will confirm or refute potential mechanisms. Our current symptom-based criteria for study entry led to small differences from placebo in therapeutic trials because similar symptoms can arise from more than one mechanism. We should subdivide our patients by the mechanisms discussed above and use as entry criteria to trials so that therapies are targeted to those who can benefit, with a potential for reducing the time and cost of developing new effective therapies. Better biomarkers more directly assessing the disturbance in function being targeted will help in this process.
